# Environmental conditions alter successional trajectories on an ephemeral resource: a field experiment with beetles in dead wood

**DOI:** 10.1007/s00442-020-04750-5

**Published:** 2020-10-07

**Authors:** Ole Petter Laksforsmo Vindstad, Tone Birkemoe, Rolf Anker Ims, Anne Sverdrup-Thygeson

**Affiliations:** 1 Department of Arctic and Marine Biology, University of Tromsø, The Arctic University of Norway, Framstredet 39, 9037 Tromsø, Norway; 2 Faculty of Environmental Sciences and Natural Resource Management, Norwegian University of Life Sciences, P.O.Box 5003, 1432 Aas, Norway

**Keywords:** Decomposition, Environmental context dependency, Facilitation, Functional species group, Saproxylic

## Abstract

Successional processes can be observed for many organisms and resources, but most studies of succession have focused on plants. A general framework has been proposed, advocating that successional patterns in species turnover are predominantly driven by competition, dispersal or abiotic limitation, and that the patterning of species accumulation over time gives clues to which process is most influential in a given system. We applied this framework to succession in communities of wood-living beetles, utilizing ephemeral resources in the form of 60 experimentally created dead aspen high stumps. High stumps were created at sun-exposed sites (high ambient temperature; favourable abiotic conditions) and shaded sites (low ambient temperature; abiotically limiting conditions). The sites were intermixed, ensuring similar dispersal opportunities. Beetle species richness and abundance were monitored with flight interception traps over four consecutive years. Consistent with predictions from the tested framework, several beetle functional groups accumulated species more slowly at the unfavourable shaded sites than at the favourable exposed sites. Species richness at the exposed sites increased rapidly to a plateau, consistent with a limiting effect of competition on community development. Similar results were obtained for beetle abundance and community structure. Part of the variance in beetle community structure was jointly explained by habitat and fungal community composition, suggesting that differences in the composition and developmental rate of fungal communities in the two habitats contributed to the observed patterns. Targeted experimental studies are now required to decisively establish what processes underlie the contrasting successional trajectories in the two environments.

**Electronic supplementary material:**

The online version of this article (10.1007/s00442-020-04750-5) contains supplementary material, which is available to authorized users.

## Introduction

Ecological successions are among the most important and well-studied processes that occur in natural ecosystems. Successions occur across a wide range of spatial and temporal scales, ranging from landscape-level processes, such as forest regrowth after fire, to the colonization and utilization of ephemeral and patchy resources, such as carrion, dung or dead wood (Anderson 2007; Weslien et al. 2011; Michaud et al. 2015). Classical botanical succession represents a series of gradual changes in ecosystem state following the appearance of uncolonized habitat (primary succession), or the disturbance of a pre-existing community (secondary succession), and proceeds towards a climax state (Walker et al. 2003). Meanwhile, succession on ephemeral resources starts with the appearance of the resource and ends with its disappearance (Michaud et al. 2015). However, successional trajectories––i.e. the patterns of change in the diversity and abundance of species in the community over time––may be shaped by similar processes in both contexts.

Anderson (2007) compared successions across 16 studies covering primary and secondary successions of plants, secondary successions of arthropods on mangrove islands and successions of arthropods on carcasses (i.e. an ephemeral resource). For systems where resources were easily available and competition was high, she found that rates of species gain (i.e. the rate at which new species were added to the community) tended to be high from the start of the succession but decline rapidly over time. These conditions were represented by secondary plant successions and primary plant successions in favourable environments, including sites at low altitudes and in front of receding glaciers. Meanwhile, in systems where abiotic conditions were harsh and facilitation was necessary for resources to become available, species gain rates were low from the start of the succession and peaked later. These conditions were represented by primary plant successions at high altitudes. For systems that were difficult to colonize––represented by arthropods on mangrove islands––gain rates showed a linear decrease or no pattern with time. Compared to species gain rates, species loss rates were generally low and showed no strong temporal patterning. Based on these findings, Anderson (2007) proposed a general theoretical framework postulating that competition, abiotic limitation and dispersal were the main processes determining the temporal rate of succession and that each process was distinguished by a characteristic temporal patterning of species accumulation.

Anderson (2007) studied a relatively limited sample of organisms and systems, and the only ephemeral resource system she considered (arthropods on carcasses) was difficult to place within the framework outlined above. Thus, additional studies are needed to assess the general validity of her conceptual framework, especially for ephemeral resources. Dead wood represents an ephemeral resource associated with a high biodiversity, especially among invertebrates and fungi (Grove 2002; Stokland et al. 2012). Dead wood is also a substrate that it is easy to manipulate experimentally and provides an excellent model system for studying succession on ephemeral resources. In the present study, we aim to apply the framework of Anderson (2007) to an ephemeral resource system consisting of beetles utilizing dead wood.

Successions are usually characterized by stages shaped by the organisms and environment in combination (Lee et al. 2006; Vanderwel et al. 2006; Weslien et al. 2011; Stokland et al. 2012; Pechal et al. 2014). These stages often correspond to functional species groups, resulting in a predictable procession of functional characteristics in the community over time (Swenson et al. 2012; Gibb et al. 2013; Pechal et al. 2014). This is also evident in successions on dead wood. Living trees have chemical defense systems that gradually wear off after tree death. Specialist wood-feeding decomposers, which are adapted to these lingering chemical defenses, may therefore be needed to attack the wood in the initial stages of decay (Stokland et al. 2012). Early stages of decay also include highly nutritious resources in the cambium, potentially causing strong resource competition, which is expected to favour specialists over generalists (Futuyma et al. 1988; Grove et al. 2011; Wende et al. 2017). As the succession proceeds and fungi colonize, the fungal community breaks down complex compounds in the wood and redistributes nitrogen and phosphorous, thereby making more nutrients available for insects (Six et al. 2019). The fungus itself also provides food for arthropods (Lawrence 1989). On the individual trunk level, the functional groups also change during decay, with fungivores generally peaking at a later stage of decay than wood-feeders (Vanderwel et al. 2006; Brunet et al. 2009; Grove et al. 2011).

**Fig. 1:**
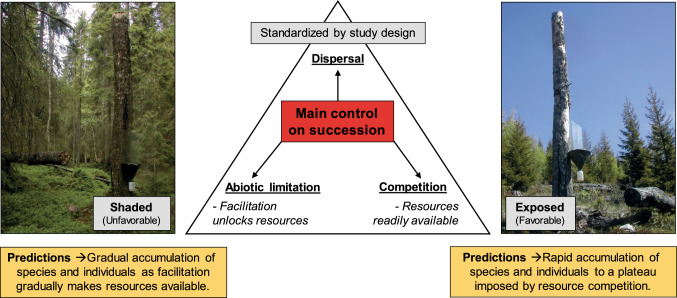
An overview of the conceptual framework of the study, inspired by Anderson (2007). Temporal patterns of beetle succession on experimentally created aspen high stumps are hypothesized to be governed by three main controls, i.e. competition, dispersal and abiotic limitation. Our study design standardizes opportunities for dispersal, while establishing a contrast between two environmental contexts where competition and abiotic limitation are expected to be the main controls. These respective environments are sun-exposed clearings, which are expected to be favourable for aspen-associated beetles, and shaded closed forest, which is expected to be unfavourable. Resources are expected to be readily available at the favourable exposed sites, while facilitative processes may be required to unlock part of the resource pool at the less favourable shaded sites. Accordingly, beetle communities at exposed sites are expected to accumulate species and individuals at a high rate from the start of the succession, until a plateau is reached due to resource competition. Meanwhile, the abiotically limited shaded sites are expected to show a more gradual accumulation of species and individuals, as facilitation gradually makes resources available. The photos show a trunk-mounted window trap on an experimental high stump in each environmental context. Photo: Anne Sverdrup-Thygeson

The goal of the present study is to characterize early successional patterns in functional groups of wood-living beetles and assess how these patterns conform to Anderson’s (2007) proposed framework of successional rates. We use experimentally created aspen high stumps as our focal ephemeral dead wood resource. Aspen represents an early successional tree in European forests, with a high diversity of aspen-associated beetle species, many of them red listed (Sverdrup-Thygeson et al. 2002; Lachat et al. 2012; Rubene et al. 2014). Canopy openness (microclimate) is a major abiotic driver of wood-living species richness in temperate forests (Seibold et al. 2016), and many aspen-associated species show strong preferences for sun-exposed wood (Sverdrup-Thygeson et al. 2002; Lachat et al. 2012; Rubene et al. 2014). Thus, we compare the succession of beetles on experimentally created dead aspen between contrasting microclimatic sites, fully sun exposed and shaded. This is expected to parallel the abiotic limitation gradient described by Anderson (2007), where competition for resources is likely to be most important at the most favourable sites (sun-exposed), whereas abiotic limitation is expected to have a greater role relative to competition in the less favourable sites (shaded) (Fig. 1). To avoid differences in dispersal limitation, which may disturb the successional trajectories between our sites, the two contrasting environments are intermixed in our experimental set-up. To start the succession, we use detonating cord to experimentally kill a sample of 60 aspen trees, thereby producing standing high stumps. Using trunk-mounted window traps on the high stumps, we compare the simultaneous development of beetle communities in the two contrasting abiotic environments during the first four years following tree death. In boreal forest, the wood is colonized by a high diversity of saproxylic species during this period, including many specialized species, and there is substantial turnover in the composition of the saproxylic community (Hammond et al. 2001; Ranius et al. 2011; Stokland et al. 2012). Thus, these first few years after tree death are well suited to comparing successional rates between different microclimatic conditions.

Based on the framework of Anderson (2007), we predict that species gain rates will be high from the start of the succession at the abiotically favourable sun-exposed sites, but quickly decrease as most species in the community become established and resource competition increases. Meanwhile, we expect maximum gain rates to occur later at the less favourable shaded sites, where facilitation by fungi and early-colonizing beetle species may be necessary for the part of the resource pool to become accessible. As we are targeting the early stages of succession, we expect species loss rates to be low relative to gain rates in both habitats. Accordingly, the exposed high stumps are expected to accumulate species faster than stumps in shaded sites. Further, if resources are indeed easily accessible at the exposed sites, we expect that beetle abundance there (i.e. the number of individuals) will increase rapidly from the start of the succession but stabilize when competition becomes limiting. Beetle communities at shaded sites, where facilitation may play a larger role in unlocking resources, are predicted to show a more gradual increase in abundance (Fig. 1). If these predictions regarding the accumulation of species and individuals in the two habitats hold true, shaded sites should also exhibit more gradual changes in beetle community structure––defined here as the relative abundances of different beetle species. With respect to beetle functional groups, we predict that host-tree specialists––which are expected to be strong competitors and well adapted to lingering chemical defenses in the dead wood––will gain species and individuals at a higher rate than generalists during the early stage of succession. We expect that this will be the case mainly for exposed sites, where competition is likely to be strongest. Wood feeders are predicted to be the most abundant during the first years of the succession, when the nutrient-rich cambium is available, while fungivores are expected to increase through time as fungi establish. The predators are expected to respond numerically to available prey.

## Material and methods

### Study area

**Fig. 2:**
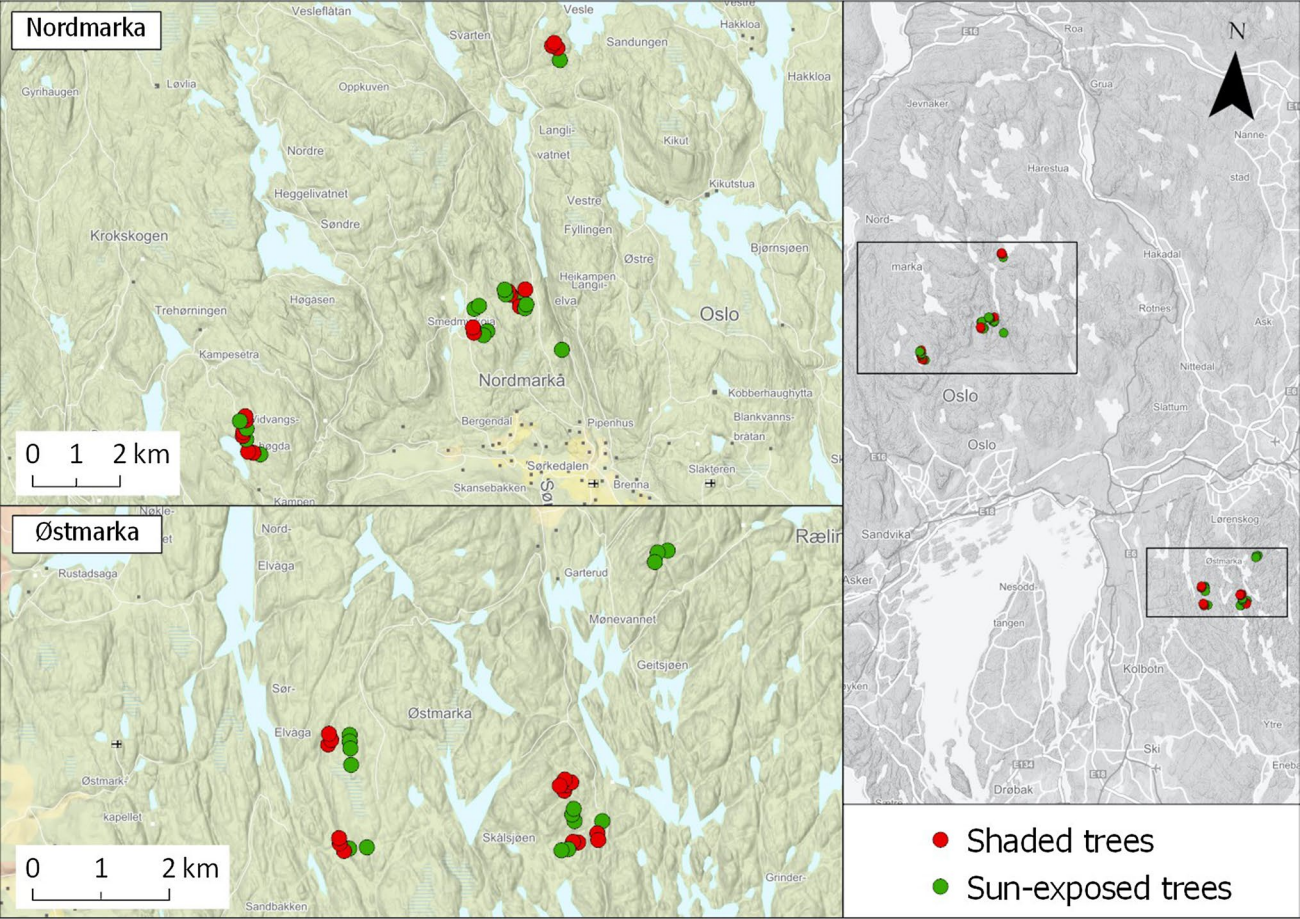
Map of the study region, showing the location of the experimental trees within the two landscapes of the study

The field study was conducted during 2001–2005 in two landscapes in the southern boreal vegetation zone (Moen 1998) in Southern Norway; Losby forest holdings in Østmarka (Lat. 55.98, Long.10.68, 150–300 masl) and Løvenskiold-Vækerø forest holdings in Nordmarka (Lat. 54.49, Long. 21.24, 200–500 masl) (Fig. 2). The dominant tree species in the both areas was Norway spruce (*Picea abies*), with Scots pine (*Pinus sylvestris*), birch (*Betula pubescens*) and aspen (*Populus tremula*) as subdominants.

Both landscapes represent modern, managed forest with average amounts of dead wood in a Norwegian context, i.e. approximately 9 m^3^/ha (Storaunet et al. 2011). We also estimated the density of dead and living aspen in both landscapes by transects covering ca 0.2% of each landscape. Østmarka landscape had twice the density of dead aspen compared to the Nordmarka landscape (4.6 versus 1.9 dead aspens/ha, 28.0 versus 11.2 living aspens/ha).

Within the two landscapes, forest stands with large deciduous trees (according to the forest inventory database) were surveyed and all aspen trees exceeding 20 cm diameter and situated at least 10 m away from the stand border were mapped with a GPS. From the resulting database of 230 trees, we randomly selected 15 trees in 2- to 4-year-old clear-cuts (representing sun exposure, Fig. 2a) and 15 trees in closed canopy forest (age 90–120 years, representing shade, Fig. 2b) within each landscape, totalling 60 aspen trees. The sun-exposed and shaded sites were mixed in the landscape (Fig. 2), and the majority of the trees belonged to different forest stands, with a minimum distance of 100 m between trees. We note that the average stand size in Norway is small, on average 1.4 ha (https://www.regjeringen.no/globalassets/upload/lmd/vedlegg/brosjyrer_veiledere_rapporter/norwegian_forests_2007.pdf). This reflects a high level of heterogeneity in both terrain and forest structure.

### Beetle sampling and creation of decaying trees

The beetle sampling commenced in spring 2001, when large (40 cm × 60 cm) trunk window traps (TWT) (Kaila et al. 1997) were mounted on the 60 trees. Each trap was mounted on a tree, facing south, and with the lower edge of the window pane 1 m above ground (Fig. 1). In the late fall of 2001, all trees were cut approximately 4 m above ground using detonating chord. The resulting logs were left in place at the base of the high stump (Fig. 1). The beetle trapping commenced in 2002–2005, operating from mid-May to mid-August in all years. The sampling years are hereafter abbreviated as years 1–4 for the four years of succession. All beetles were identified and categorized into functional groups (see below), based on literature (Hansen et al. 1908; Palm 1959), a database compiled by Dahlberg and Stokland (Dahlberg et al. 2004) and the Norwegian Red List Database (https://www.biodiversity.no/Article.aspx?m=39&amid=1864). We first grouped the beetles into two subgroups according to host tree affinity, namely aspen specialists (species mainly associated with aspen) and aspen generalists (species associated with aspen and other three species). Species not associated with aspen were excluded from the analysis. Thereafter, all aspen-associated beetles were grouped into trophic guilds, namely fungivores which feed on fungus or fungus-infested wood, wood-feeders which feed on dead wood, predators that attack other wood-living invertebrates, and omnivorous or saprophagous species that did not fit into the other categories.

A study of insect succession in dead wood should predominantly include species that actually breed in the wood habitat. Emergence traps collect species emerging from the wood but simultaneously prevent new colonization and interrupt succession (Lindhe et al. 2004; Birkemoe et al. 2015). Trunk window traps, on the other hand, represent a non-destructive sampling option with well-known sampling efficiency: (1) they catch the same proportion of aspen-associated species as emergence traps from aspen dead wood (Birkemoe and Sverdrup-Thygeson 2015); (2) the catches of wood-boring beetle species correspond with the presence of species-specific exit holes on the same aspen trunks (Sverdrup-Thygeson and Birkemoe 2009); (3) the total catches may correlate with the number of exit holes on the same aspen trunk (as has been shown for the species *Rusticoclytus rusticus*) (Sverdrup-Thygeson and Birkemoe 2009) and (4) they reflect the same main responses to environmental gradients in beetle communities as other sampling methods (Sverdrup-Thygeson et al. 2009; Müller et al. 2015). With respect to the comparison of shaded and sun-exposed habitats undertaken in the current study, sun exposure could elevate temperature and thus lead to increases in beetle activity. This might cause some bias towards higher estimates of beetle abundance and species richness at exposed sites. However, we do not expect this to produce biases with respect to the temporal patterns of beetle succession (i.e. the year-to-year variation within environments) that are the focus of our predictions.

To provide a measure of the amount of dead wood in the immediate vicinity of the high stumps, the number of standing dead aspen trees were recorded in a 30 m radius around the stumps in 2002. Seventy percent of the high stumps had no standing dead aspen within 30 m, and only five stumps had two or more dead aspens. A zero-inflated log-linear model showed no evidence for significant differences between shaded and sun-exposed forest sites in the amount of dead aspen close to the high stumps (*P* = 0.49).

In 2005 we surveyed the high stumps for occurrence of fungi easily recognizable by their fruit bodies (*Chondrostereum purpureum*, *Phellinus tremulae* and *Trametes* sp.) and slime molds, and calculated the diversity (denoted fungal diversity) based on this. Surveys of fruiting bodies do not reveal the year of colonization, but most fungi will be present in the wood for some time before developing their reproductive organs (Boddy 2001). Thus, even though our survey was conducted in 2005, we expect it to reflect the community of fungi present during the trapping years.

### Statistical analyses

The changes that occur in a community during succession can be described in terms of the number of individuals (abundance) or species that are present. Abundance represents the numerical response of the community to the resource that becomes available at the start of the succession, while species richness is a product of species colonizations and extinctions. Because abundance and richness both represent important descriptors of the functional state of a community, and both convey information about the speed of community changes during succession, we consider temporal patterns in both richness and abundance in our analysis.

Following Anderson (2007), we partitioned changes in species richness into species gain rates (colonizations) and species loss rates (extinctions), denoted *G*_p_ and *L*_p_, respectively. These measures are defined as follows:

$$G_{{\text{P}}} = \frac{G}{{\left( \frac{1}{2} \right)\left[ {S_{t1} + S_{t2} } \right]}},$$

$$L_{{\text{p}}} = \frac{L}{{\left( \frac{1}{2} \right)\left[ {S_{t1} + S_{t2} } \right]}},$$

where *S*_*t*1_ and *S*_*t*2_ are species richness at times *t*1 and *t*2, respectively, and *G* and *L* are the number of species gained and lost, respectively, during the time interval between *t*1 and *t*2. *G*_p_ and *L*_p_ thus measure the magnitude of species losses and gains, relative to the existing community, respectively. Note that a temporary (non-permanent) loss of a given species was not counted as an extinction when calculating *G* and *L*. Hence, a species was only considered to have been gained by the community in a given year if it had been absent in all previous years and was only considered to have been lost if it was absent in all following years.

Patterns of gain and loss rates, species richness and abundance for the different functional groups of beetles were analysed with generalized linear mixed models, using the glmer and lmer functions in the lme4 library in R (Bates et al. 2015). The predictors time and environmental conditions were taken as fixed factors in the models. Environmental conditions was defined as a categorical predictor with the levels ‘sun-exposed’ or ‘shaded’, while time was taken as a categorical predictor with years 1–4 as levels in the models for species richness and abundance, and the time intervals 1–2, 2–3 and 3–4 as levels in the models for gain and loss rates. Because our core predictions concerned differences in the temporal patterning of succession between exposed and shaded environments, the interaction between time and environment was of crucial importance to the analysis. For all models, we used a likelihood ratio test to assess the overall significance of the interaction term. If the interaction was significant, we estimated the interaction term and derived predictions from a model including the interaction. This allowed us to infer how the temporal patterning of succession differed between exposed and shaded conditions, up an above the additive main effects of time and environment, and whether the environment-specific successional patterns matched our predictions. Moreover, to assess the difference between shaded and exposed conditions within each year/interval, we used pairwise comparisons implemented with the functions cftest and glht in the multcomp library in R. The effect of landscape (Østmarka or Nordmarka) was not of explicit interest to us and was therefore included only as a random factor (to account for differences in the amount of aspen and other unknown sources of variation associated with landscape). The identity of individual trees was also taken as a random factor, nested within landscape. Species richness and abundance were modelled with a negative binomial error distribution, to account for highly aggregated counts, while species gain and loss rates were modelled with normally distributed errors. Parameter estimates, predicted values and pairwise comparisons for all mixed models are given in online resource 1 tables S1–3 (species gain rates), S4–6 (species loss rates), S7–9 (species richness) and S10–12 (abundance).

In addition to our prediction that the temporal patterning of succession would diverge between environments within beetle functional groups, we also expected that aspen specialist beetles would accumulate species and individuals more rapidly than generalists from the start of the succession at exposed sites. To test this prediction, we fitted models where functional group (‘specialist’ or ‘generalist’) was included as a third predictor, in addition to year and environment. As the prediction concerned the start of the succession, the models were fitted only for years 1 and 2. In these models, a more rapid increase in specialists would be manifested as a significant positive three-way interaction between environment, year and functional group. Parameter estimates and predictions from these models are presented in online resource 1 tables S13 and S14.

Temporal autocorrelation can be a problem in studies dealing with repeated measurements on the same sampling units (in this case the high stumps). To address this problem, we examined plots of residuals against time at the level of the individual sampling units for all mixed models. In the great majority of cases, these plots displayed a random scatter of residuals, with no indication of temporal patterning. This suggests that the year effect adequately accounted for the temporal structuring in the data and that residual temporal autocorrelation was not a major issue.

Redundancy analysis (RDA, performed with the rda function in the vegan package in R) was used to investigate changes in beetle community structure throughout the years of the succession, depending on environmental conditions, and to identify species that were important in driving these changes. Prior to RDA, all beetle counts were Hellinger transformed (Legendre et al. 2001). Owing to relatively small numbers of individuals in many of our functional groups, we included aspen specialists and generalists and all trophic guilds in a single RDA model. Year (1–4), environmental conditions (sun exposed or shaded) and their interaction were taken as categorical predictors, while landscape was used as a conditional variable. We tested the marginal significance of the predictors with permutation tests (anova.cca function in the vegan package, run with 1000 permutations). The results from the RDAs were displayed in triplots, using type II scaling of the ordination scores (Legendre et al. 2012).

We used variance partitioning (the varpart function in the vegan package) to estimate how much of the variance in beetle community structure was explained by the predictors year (1–4) and environmental conditions (sun exposed or shaded). We also investigated if fungal diversity at the level of individual trees accounted for additional variance by adding this variable as an additional predictor. Landscape (Østmarka or Nordmarka) was also tried as a predictor, but its overall contribution to explaining variance in the beetle community was only 1%. Landscape was therefore omitted to simplify the model. The results of the variance partitioning were visualized using venn diagrams, drawn with the venneuler package in R.

## Results

10,157 aspen-associated saproxylic beetle individuals from 184 species and 42 families were captured during the study. 1249 individuals from 17 species were classified as aspen-associated host-tree specialists, while 8908 individuals from 167 species were classified as aspen-associated generalists. 1630 individuals from 22 species were classified as wood-feeders, 3961 individuals from 73 species as fungivores and 3318 individuals from 75 species as predators. Finally, 1248 individuals from 14 species fell into the omnivore/saprophage trophic group.

### Successional trajectories in the gain, loss and richness of beetle species

**Fig. 3:**
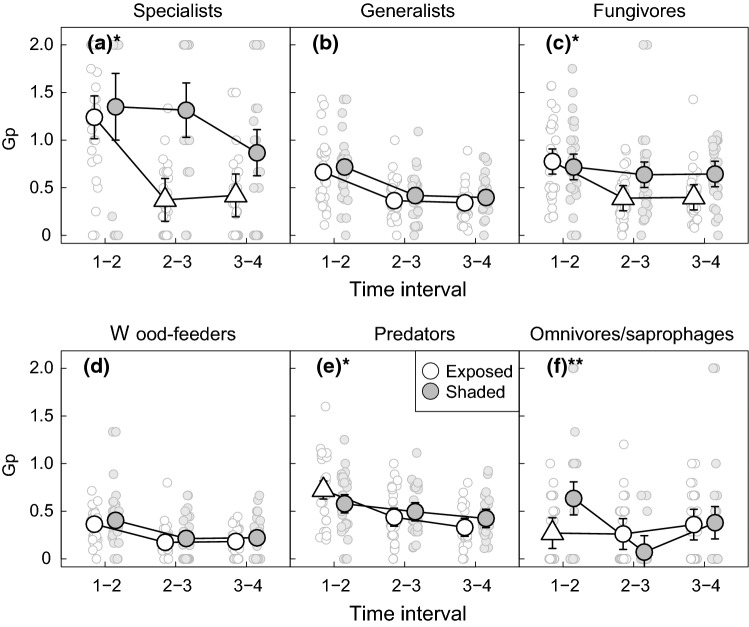
Variation in the species gain rates (Gp) of host-tree specialists (**a**), host-tree generalists (**b**), fungivores (**c**), wood-feeders (**d**), predators (**e**) and omnivores/saprophages (**f**) across the 4 years of succession in sun-exposed (white symbols) and shaded (grey symbols) sites. Large symbols represent predicted gain rates from linear mixed models for each combination of time interval and sun exposure. Error bars represent 95% confidence intervals. Predictions for exposed sites are represented by a triangle for time intervals where pairwise comparisons revealed a statistically significant difference in gain rates between exposed and shaded sites. Stars in the lettering of the panels indicate the statistical significance of the interaction between sun exposure and time interval. ***P <  = 0.001, **P <  = 0.01, *P <  = 0.05. Small symbols represent observed values for individual traps

**Fig. 4:**
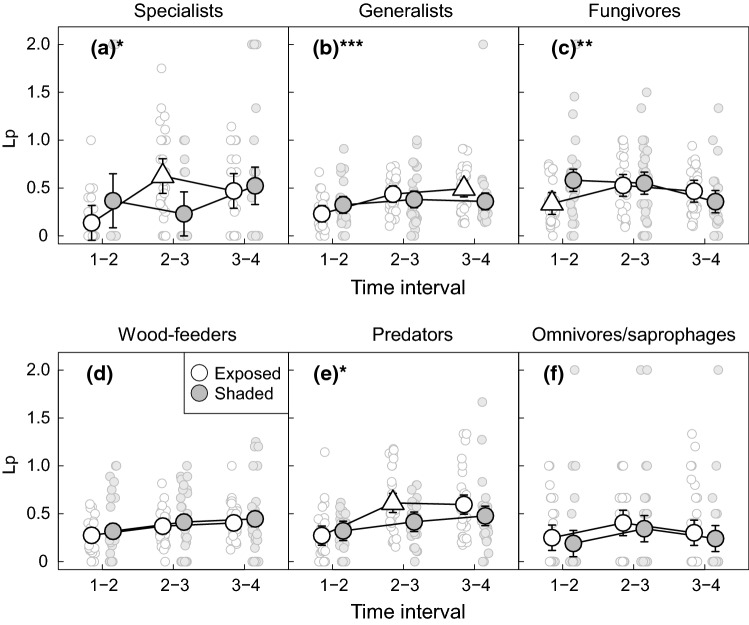
Variation in the species loss rates (Lp) of host-tree specialists (**a**), host-tree generalists (**b**), fungivores (**c**) wood-feeders (**d**), predators (**e**) and omnivores/saprophages (**f**) across the 4 years of succession in sun-exposed (white symbols) and shaded (grey symbols) sites. Large symbols represent predicted loss rates from linear mixed models for each combination of time interval and sun exposure. Error bars represent 95% confidence intervals. Predictions for exposed sites are represented by a triangle for time intervals where pairwise comparisons revealed a statistically significant difference in loss rates between exposed and shaded sites. Stars in the lettering of the panels indicate the statistical significance of the interaction between sun exposure and time interval. ***P <  = 0.001. **P <  = 0.01. *P <  = 0.05. Small symbols represent observed values for individual traps

Three beetle functional groups conformed to our prediction that species gain rates would peak at the start of the succession at the exposed sites. Specifically, aspen specialists, fungivores and predators showed gain rates that were high for interval 1–2 and thereafter declined (Fig. 3, table S2). Meanwhile, the gain rates of these groups in the shade remained more stable throughout the succession. This resulted in significant interactions between time and environment for the gain rates of all three groups, and significant pairwise differences between gain rates in shaded and exposed conditions for intervals 2–3 and 3–4 for specialists and fungivores, and interval 1–2 for predators (Fig. 3, Tables S1 and S3). Meanwhile, the rates of species loss at exposed sites showed the opposite temporal pattern of the gain rates for all three groups, with loss rates being low for interval 1–2 and thereafter increasing (Fig. 4, table S5). The loss rates of these groups in the shade did not show consistent temporal patterns but were generally somewhat more stable than loss rates at exposed sites, resulting in significant time × environment interactions for all three groups (Table S4).

**Fig. 5:**
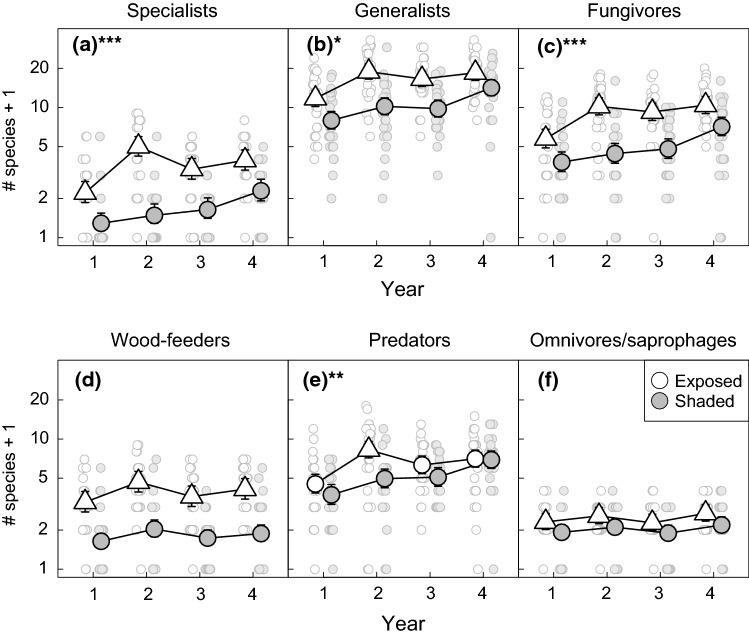
Variation in the species richness of host-tree specialists (**a**), host-tree generalists (**b**), fungivores (**c**) wood-feeders (**d**), predators (**e**) and omnivores/saprophages (**f**) across the 4 years of succession in sun-exposed (white symbols) and shaded (grey symbols) sites. Large symbols represent predicted species richness from negative binomial mixed models for each combination of year and sun exposure. Error bars represent 95% confidence intervals. Predictions for exposed sites are represented by a triangle in years where pairwise comparisons revealed a statistically significant difference in species richness between exposed and shaded sites. Stars in the lettering of the panels indicate the statistical significance of the interaction between sun exposure and year. ***P <  = 0.001, **P <  = 0.01. *P <  = 0.05. Small symbols represent observed values for individual traps

The early peak of species gain rates and the subsequent increase of loss rates at the exposed sites caused the species richness of both specialists, fungivores and predators to increase abruptly from years 1 to 2 at these sites and thereafter stabilize or even decline slightly (Fig. 5, tables S8). Meanwhile, the species richness of these groups in the shade showed an increase that was either gradual (specialists and predators) or delayed to year 4 (fungivores). These patterns caused the pairwise differences in species richness between environments to peak in year 2 for all three functional groups (Table S9). The pairwise differences then declined over the two last years, as the species richness at the shaded sites gradually approached that of the exposed sites. As a result, the time × environment interactions were highly significant for all three groups (Table S7). Thus, the results for specialists, fungivores and predators clearly supported our prediction that high stumps in exposed sites would accumulate species more rapidly than stumps in the shade.

The temporal patterns of species gain in the remaining three beetle functional groups (aspen generalists, wood-feeders and omnivores/saprophages) did not conform to our predictions. Specifically, the patterning of gain rates at exposed and shaded sites was so similar that the time × environment interaction was non-significant for generalists and wood-feeders (Fig. 3, Tables S1 and S2). Meanwhile, a significant interaction was required to describe a higher rate of species gain at shaded than exposed sites for interval 1–2 in omnivores/saprophages (i.e. the opposite of the predicted pattern). Loss rates were also very similar at exposed and shaded sites for wood-feeders and omnivores/saprophages but were somewhat higher at exposed than shaded sites during interval 3–4 for generalists (Fig. 4, table S6). These patterns of species gain and loss resulted in very similar temporal patterns in species richness at exposed and shaded sites for wood-feeders and omnivores/saprophages (non-significant time × environment interaction), while a significant interaction was required to describe a somewhat higher increase in species richness at exposed than shaded sites from years 1 to 2 in generalists (Fig. 5, table S7).

The comparison of species accumulation between specialist and generalist beetles during years 1 and 2 was only partly congruent with our prediction that specialist beetles would accumulate species more rapidly than generalists during the initial phase of succession. Based on the mixed model including both functional groups, predicted specialist species richness increased by a factor of 3.31 between years 1 and 2, while generalist richness increased by a factor of 1.66 (Table S13). However, this difference between groups was not large enough to produce a significant three-way interaction between environment, year and group, thus yielding no strong statistical evidence for faster species accumulation in specialists (Table S14).

Regardless of whether the temporal patterning of species richness differed between exposed and shaded sites, the absolute number of species was generally higher at exposed sites throughout the study period for all beetle functional groups (Fig. 5, table S8). Hence, the pairwise differences in species richness between environments within years were significant in nearly all cases (Table S9). The only exception was the predators, where species richness was significantly higher at exposed than shaded sites only in year 2. The consistently higher species richness at the exposed sites supports our assumption that sun-exposed sites are most favourable for aspen-associated beetles.

### Successional trajectories in beetle abundance

**Fig. 6:**
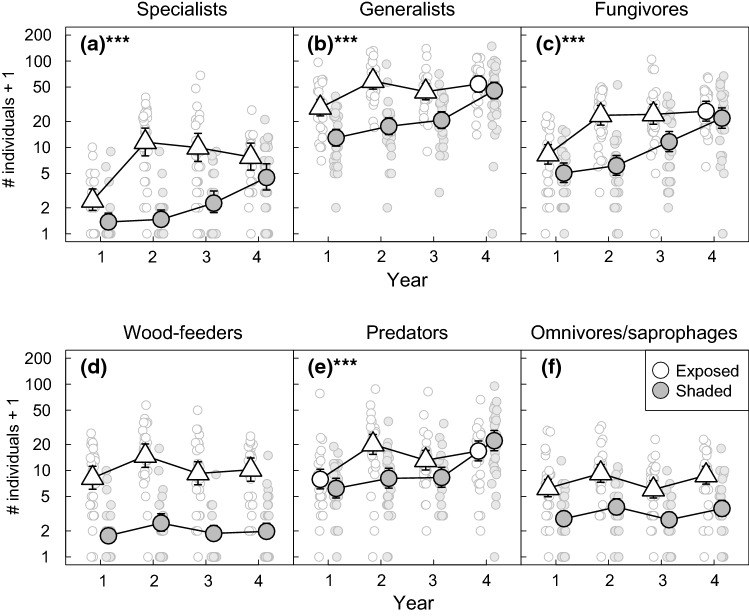
Variation in the abundance of host-tree specialists (**a**), host-tree generalists (**b**), fungivores (**c**) wood-feeders (**d**), predators (**e**) and omnivores/saprophages (**f**) across the 4 years of succession in sun-exposed (white symbols) and shaded (grey symbols) sites. Large symbols represent predicted abundances from negative binomial mixed models for each combination of year and sun exposure. Error bars represent 95% confidence intervals. Predictions for exposed sites are represented by a triangle in years where pairwise comparisons revealed a statistically significant difference in abundance between exposed and shaded sites. Stars in the lettering of the panels indicate the statistical significance of the interaction between sun exposure and year. ***P <  = 0.001. **P <  = 0.01. *P <  = 0.05. Small symbols represent observed values for individual traps

In general, the successional trends in beetle abundance mirrored the temporal patterns in species richness and supported our prediction that abundance should increase rapidly to a plateau at exposed sites but increase more gradually in the shade (Fig. 6, Table S11). This predicted pattern was observed for aspen specialists, generalists, fungivores and predators, and resulted in highly significant time × environment interactions for all these groups (Table S10). As was the case for species richness, the pairwise differences in abundance between exposed and shaded sites for these groups also peaked in year 2 and thereafter declined, as abundance in the shade gradually approached the same levels as at the exposed sites (Table S12). The most notable difference between the results for abundance and species richness concerned the comparative increase in aspen specialists and generalists during the first two years of succession. Based on the mixed model including both functional groups, predicted specialist abundance increased by a factor of 7.56 between years 1 and 2, while generalist abundance increased only by a factor of 2.10 (Table S13). This produced a highly significant three-way interaction between environment, year and functional group, thus supporting our prediction that specialists would increase more rapidly in abundance than generalists (Table S14).

Successional trends in the abundance of individual beetle species varied considerably, independently of the major trends for each trophic group (Fig. S1 and S2). Roughly a dozen common fungivorous species and about half as many predators showed temporal trends in abundance throughout the study period. Meanwhile, only two abundant wood-feeders showed evidence for successional changes in abundance, namely the two aspen specialists *Rusticoclytus rusticus and Ptilinus fuscus*. Both of these species occurred almost exclusively at the sun-exposed sites.

### Successional trajectories in beetle community structure

**Fig. 7:**
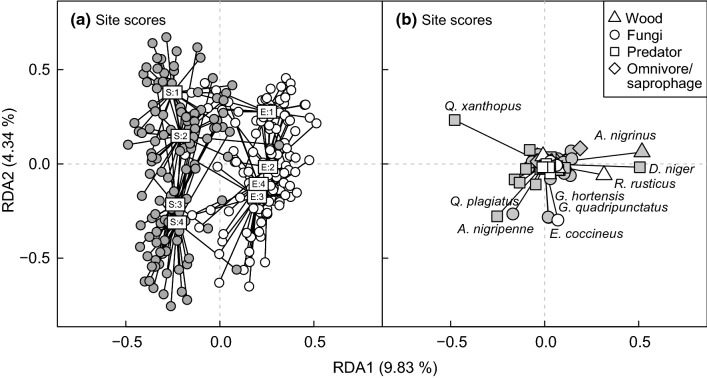
Triplots for all wood-living beetles based on RDAs with the predictors year environmental conditions and their interaction. Left panel (**a**): scores for individual traps. White and grey points represent sun-exposed and shaded sites, respectively. The boxes mark the position of the centroids for each combination of year and sun exposure. 1–4 = 2002–2005. *E* sun exposed, *S* shaded. The lines protruding from each centroid join all of the trap scores for the relevant combination of year and sun exposure. Right panel (**b**): scores for individual beetle species. Species with ordination scores with an absolute value larger than 0.2 are named in the plots. Species that are specialized on dead aspen are indicated by white points in the species panel

The RDA model revealed strong spatiotemporal structuring in beetle community structure, with sun-exposed and shaded sites being clearly distinguished along the first RDA axis, while years were distinguished along the second axis (Fig. 7a). However, the temporal patterning in community structure differed between shaded and exposed sites, resulting in a significant time × environment interaction (permutation-based ANOVA: *F* = 7.10, *DF* = 7, 229, *P* < 0.001). This interaction was required to describe a pattern that was well in accordance with our predictions, namely that beetle community structure should exhibit more gradual change at shaded than exposed sites. Specifically, the largest changes in community structure happened from the first to the second year at sun-exposed sites, whereas substantial changes occurred both between years 1 and 2, and between years 2 and 3 at the shaded sites.

The species scores from the RDA model showed that the temporal patterns in beetle community structure were mainly driven by aspen specialist fungivores (*Endomychus coccineus*, *Glischrochilus quadripunctatus*, *G. hortensis* and *Agathidium nigripenne)* and one aspen generalist predator (*Quedius plagiatus*) (Fig. 7b). All of these species showed positive temporal trends in abundance in both environments (Fig. S1 and S2). The separation between sun-exposed and shaded sites was mainly driven by wood-feeding species (*Rusticoclytus rusticus* and *Ampedus nigrinus*) and one predator (*Dasystes niger*), all of which were most abundant at the sun-exposed sites. The predator *Q. xanthopus*, which was most abundant in the shade, also made an important contribution to the distinction between sun-exposed and shaded sites. With the exception of the aspen specialist *R. rusticus*, which peaked in year 2, none of these remaining aspen generalist species showed temporal trends in abundance (Fig. S1 and S2).

**Fig. 8:**
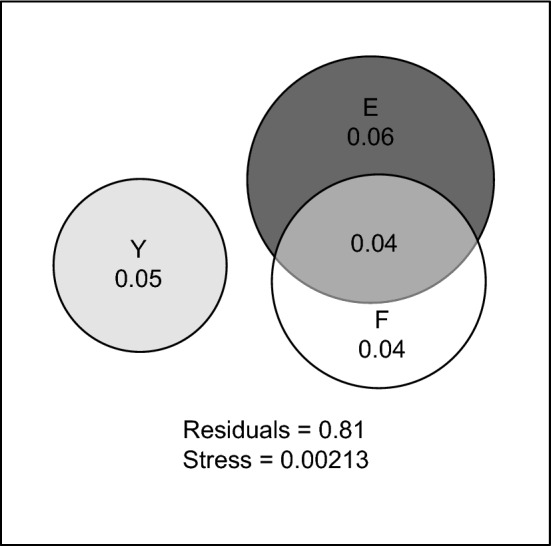
Venn diagram illustrating the contributions of environmental conditions (*E*), year (*Y*) and fungal diversity (*F*) to explaining variance in the community structure of all wood-living beetles. The size of the circles represents the amount of variance explained by the predictors relative to each other. The proportion of the total variance explained by each predictor is indicated on the circles. Overlapping areas represent variance that is collectively explained by two predictors. The residuals are the proportion of the variance in beetle community structure that is left unexplained by the predictors. The value of the venneuler stress statistic (Wilkinson 2012) is below the 0.01 threshold of *S*_0_._01_ = 0.056. This indicates that the venn diagram adequately fits the data

Year and environmental conditions uniquely explained 5% and 6%, respectively, of the variance in beetle community structure, while fungal diversity uniquely explained another 4% of the variance (Fig. 8). Moreover, the environment and fungal diversity collectively accounted for an additional 4% of the variance, suggesting that the differences in beetle community structure between sun-exposed and shaded wood were partly explained by differences in fungal diversity between the two environments.

## Discussion

Successional processes are major components of ecosystem dynamics and occur across a wide range of communities, resources and environmental conditions (Begon et al. 2006). It is therefore encouraging that the successional patterns we have documented for beetles on dead wood were at least partly congruent with predictions based on successions in plants and arthropods on other resources (Anderson 2007). Specifically, multiple lines of evidence supported the prediction that successional rates should be higher (i.e. faster) in the abiotically favourable sun-exposed sites than in the less favourable shaded sites. For both host-tree specialists and generalists, as well as fungivores and predators, species richness increased rapidly from year 1 to 2 in the exposed sites, while richness at the shaded sites increased more gradually over the course of the succession. The pattern in the exposed sites was explained by high rates of species gain and low rates of species loss in the first year of succession––a result that corresponds well with the gain and loss rates that Anderson (2007) obtained for the early stages of succession in favourable habitats for both plants and arthropods. A higher rate of succession in the favourable habitat was also evidenced in the overall structure of the beetle community (i.e. the RDA model), which changed predominantly from year 1 to 2 at the exposed sites but showed a more gradual temporal change in the shade. Finally, the abundance of specialists, generalists and fungivores increased rapidly from year 1 to 2 in exposed sites but showed a more gradual increase in the shade. Thus, the theoretically founded expectation of higher successional rates in the favourable habitat was borne out across multiple metrics of change and several functional groups.

The mechanisms underlying the contrasting successional patterns in exposed and shaded forest cannot be inferred directly from our data. However, Anderson (2007) suggested that competition for resources will replace abiotic limitation as the main control on successional rates when environmental conditions become less severe. Several beetle groups showed patterns that are compatible with competition at the exposed sites, in the sense that both richness and abundance increased rapidly from year 1 to 2 and subsequently stabilized or even decreased. This apparent saturation of the community in terms of both diversity and abundance would certainly be consistent with competition for limited resources. Anderson (2007) made a similar observation for successional time series of plants. It is also noteworthy that specialist beetles––which are expected to be strong competitors––increased more rapidly in abundance from year 1 to 2 than generalists. Competition is important in succession on other ephemeral resources, such as carrion and dung, where most nutrients are readily available from the start (Kneidel 1984; Smith et al. 1997; Morton et al. 2000; Horgan 2005; Vernes et al. 2005), but studies of competition among wood-living insects are few. However, colonization by bark and longhorn beetles has been shown to limit other cambium-feeding species (Paine et al. 1981; Schroeder et al. 1994; Brin et al. 2018). Thus, it is plausible that competition played a part also in our system, although this will have to be confirmed by experimental studies.

Anderson (2007) observed that plant communities in habitats with severe abiotic conditions (high elevations) displayed species gain rates that were low early in the succession and peaked only after considerable time had passed. Our results for beetle succession in shaded sites––which we assumed to be abiotically less favourable than exposed sites––did not display a similar pattern. Rather, shaded sites often exhibited species gain rates that were more temporally stable than the exposed sites, resulting in a more gradual accumulation of species. This may indicate that dead aspen in shaded sites is initially suitable for colonization only by a subset of the species that could theoretically utilize the dead wood resource. In contrast, most species appeared to have colonized the exposed sites already by the second year of the succession. In plant successions, delayed accumulation of species in less favourable conditions are often explained by early colonizers facilitating conditions for later species (Mcauliffe 1988; Cornell et al. 2014). Facilitation also occurs in deadwood ecosystems, for example, when the establishment of fungi provides new resources for wood-living beetles and wood-boring beetles promote opportunities for other beetles in the galleries (Allison et al. 2001; Harrington 2005; Weslien et al. 2011). In our study, more variation in species composition could be explained by the combined effect of fungi species richness and environmental conditions combined than the environment alone. This suggests that the availability of fungal resources is a potential determinant of differences in beetle successional trajectories between habitats. Beetle–fungi interactions is emerging as an important research frontier in the study of saproxylic organisms and wood degradation (Birkemoe et al. 2018; Jacobsen et al. 2018a, b; Biedermann et al. 2019; Six et al. 2019) and elucidating the effects of the abiotic environment on these interactions is an important challenge for the future (see also below).

Another difference between our results and those of Anderson (2007) concerns species loss rates. Anderson found that loss rates rarely showed clear temporal patterns and were typically low relative to gain rates from the start of the succession. Loss rates were indeed low compared to gain rates at exposed sites in the first year of succession for specialists, generalists, fungivores and predators (see above), but loss and gain rates were generally of similar magnitude in our data. High loss rates could to some extent be an artefact of working with a community with many rare species, because rare species could tend to disappear from the dataset due to imperfect sampling rather than true extinction. However, rare species could also inflate gain rates, as a rare species that has been present for some time, but eluded trapping would count as a colonization in the year that it is first sampled. The high loss rates in our data may also have a biological basis, as dead wood undergoes rapid changes in structure and quality during the early stages of decay (Stokland et al. 2012). The dead wood resource may therefore become unsuitable for many species relatively quickly, resulting in many extinctions even over a period of just four years. Stokland et al. (2012) described four broad phases of sucession of invertebrates and fungi on dead spruce in boreal forest, each characterized by distinct assemblages of species. Phases 1 and 2 occur within the first ten years after tree death, with species diversity in the second phase peaking already in year 5. These pahses include the consumption of the inner bark and the establishment of early-colonizing fungi. Similar phases occur in aspen, but aspen wood decays more rapidly than spruce, so that our 4-year time series probably covered most of the two initial phases. Thus, we would expect to see considerable turnover of saproxylic species even within the relatively limited timeframe of our study. Indeed, a rapid turnover of saproxylic beetle species in aspen wood during the first few years after tree death has previously been demonstrated using both window traps and insect rearing from wood samples (Hammond et al. 2001; Ranius et al. 2011).

The successional patterns we observed for trophic functional groups of beetles were only partly consistent with our predictions. Wood-feeders were expected to be the first group to increase in abundance, independently of environmental conditions, but showed no numerical response to our experimental snags in shaded sites. Further, only two aspen specialist wood-feeding species responded numerically to the high stumps at the exposed sites (Fig. S1), while wood-feeders as a group showed no overall increase in abundance. These observations highlight two important points regarding aspen as a resource for wood-feeding beetles. First aspen-associated wood-feeders are typically specialized on sun-exposed wood, and our results suggest that this specialization is strong enough to preclude the utilization of dead wood resources in shaded conditions. Hence, sun exposure may act as an environmental filter that excludes aspen-associated wood-feeders from some habitats. This corroborates a series of other studies that have highlighted the importance of sun-exposed wood for aspen-associated saproxylic beetles (Martikainen 2001; Sverdrup-Thygeson et al. 2002; Lindhe et al. 2004; Ranius et al. 2011; Schroeder et al. 2011). Second, our results indicate that utilization of aspen wood in the early stages of decay may require a high degree of specialization on this host tree species, perhaps owing to lingering defensive chemicals in the wood (Stokland et al. 2012).

Another unexpected finding is that the abundance of several fungivorous species peaked simultaneously with the two most abundant wood-feeders in the second year of succession in the sun-exposed sites (Fig S1 and S2). This indicates that some fungi establish rapidly on dead aspen under favourable conditions, and thereby provide fungivores with resources from an early stage in the succession. The optimal temperature for the growth of wood-living saprotrophic fungi is in the range of 20–30 °C (Boddy 1983). Sun exposure will frequently assure this temperature, whereas temperatures in shaded forest are often lower (median temperature in Oslo in July is 16.4 °C, www.met.no), thereby delaying fungal growth. Remaining bark and sap pressure from the roots may ensure favourable humidity during the first years in both environments, counteracting loss of moisture in the sun-exposed sites. This mechanism was also thought to contribute to the higher number of fungivore beetles on sun-exposed snags than logs found by Gibb et al. (2013). In parallel, the higher number of insects in the sun-exposed sites also increases the number of potential fungal vectoring incidences (Strid et al. 2014; Jacobsen et al. 2015, 2017), which may further enhance fungi development on the exposed vs shaded high stumps. Whatever the cause, an initial delay in fungal growth caused by abiotic conditions may be the most important factor differentiating successional trajectories in sun-exposed and shaded sites, in addition to the differences in the wood-feeder fauna.

Predaceous beetles as a group showed no clear temporal trends in abundance in exposed forest but exhibited a marked increase in shaded forest in the final year of the succession. The increase in predator density was thus somewhat delayed compared to the increase in fungivores (i.e. potential prey). This time lag would be compatible with a reproductive response to increasing prey density in the predators. However, individual predator species showed more variable temporal patterns, with several species increasing in abundance in both shaded and exposed forest sites already in year 2 (Fig. S2). This suggests that some predators also exhibited aggregative responses, with predators being attracted to the stumps when their prey increased. Wood-living predators may be attracted to kairomones emitted by their prey. This is well documented for *Rhizophagus* species (Gregoire et al. 1991; Meurisse et al. 2008; Wehnert et al. 2012), which were among the predators showing the clearest numerical responses in the present study.

## Conclusion

We have demonstrated that the successional trajectories of wood-living beetles in aspen are partly congruent with predictions from a general theoretical framework, derived based on succession in other organism groups and habitats/resources. Specifically, beetle communities at favourable sun-exposed sites generally accumulate both species and individuals more rapidly than communities at less favourable shaded sites. However, these successional changes also decelerate more rapidly at exposed sites. These trajectories are compatible with competition and abiotic limitation, respectively, as the main controls on successional rates. Differences in the composition and developmental rate of fungal communities in the two habitats could be one factor explaining these patterns. It may also be hypothesized that facilitative processes play an important role in the shade. However, experimental studies are needed to provide decisive proof of the mechanisms underlying the patterns that we have documented.

## Electronic supplementary material

Below is the link to the electronic supplementary material.

Supplementary file1 (DOCX 1074 kb)

## Data Availability

The datasets generated during and/or analysed during the current study are available in the DRYAD digital repository, 10.5061/dryad.wh70rxwjr.
