# Supplementary material for: Environmental conditions alter successional trajectories on an ephemeral resource: a field experiment with beetles in dead wood
Source: Oecologia. 2020 Oct 7;194(1):205–19. doi: 10.1007/s00442-020-04750-5 (PMC7561563; doi:10.1007/s00442-020-04750-5)
Supplement: Supplementary file 1 — Supplementary file1 (DOCX 1074 kb) [file 442_2020_4750_MOESM1_ESM.docx]

**Online resource 1. Supplementary tables and figures**

**Table S1.** Coefficients for the fixed effects in the selected linear mixed models taking beetle species gain rate (see main text for definition) as the response, and the factors time interval (1-2, 2-3 and 3-4) and environmental conditions (sun-exposed or shaded) as predictors. There is a separate model for the following beetle functional groups: a) aspen specialists b) aspen generalists c) fungivores d) wood-feeders e) predators f) Saprophages/omnivores. The intercept in all models represents shaded high stumps for the time interval 1-2. Ninety-five % confidence intervals are given in brackets, and terms that are statistically significant on the 0.05 level are highlighted in bold. *** = P <= 0.001. ** = P <= 0.01. * = P <= 0.05. Note that the time × environment interaction was not significant for b) and d) and was therefore dropped from these models.

| Parameter | a) Aspen specialists | b) Aspen generalists |
| --- | --- | --- |
| Intercept | **1.35 [1.00, 1.70]***** | **0.72 [0.64, 0.80]***** |
| Envir cond (Sun) | -0.11 [-0.53, 0.31] | -0.05 [-0.13, 0.03] |
| Interval (2-3) | -0.04 [-0.49, 0.42] | **-0.30 [-0.39, -0.21]***** |
| Interval (3-4) | **-0.48 [-0.91, -0.06]*** | **-0.32 [-0.41, -0.23]***** |
| Interval (2-3) × Envir cond (Sun) | **-0.83 [-1.38, -0.28]**** | **-** |
| Interval (3-4) × Envir cond (Sun) | -0.34 [-0.87, 0.19] | **-** |
|  | c) Fungivores | d) Wood-Feeders |
| Intercept | **0.72 [0.58, 0.85]***** | **0.40 [0.34, 0.47]***** |
| Envir cond (Sun) | 0.06 [-0.13, 0.24] | -0.04 [-0.10, 0.02] |
| Interval (2-3) | -0.08 [-0.27, 0.11] | **-0.19 [-0.27, -0.12]***** |
| Interval (3-4) | -0.07 [-0.26, 0.12] | **-0.18 [-0.26, -0.11]***** |
| Interval (2-3) × Envir cond (Sun) | **-0.30 [-0.57, -0.04]*** | **-** |
| Interval (3-4) × Envir cond (Sun) | **-0.30 [-0.57, -0.03]*** | - |
|  | e) Predators | f) Omnivores/saprophages |
| Intercept | **0.58 [0.48, 0.67]***** | **0.63 [0.46 ,0.81]***** |
| Envir cond (Sun) | **0.14 [0.01, 0.28]*** | **-0.36 [-0.60, -0.13]**** |
| Interval (2-3) | -0.08 [-0.22, 0.05] | **-0.56 [-0.81, -0.32]***** |
| Interval (3-4) | **-0.15 [-0.29, -0.02]*** | **-0.26 [-0.50, -0.01]*** |
| Interval (2-3) × Envir cond (Sun) | **-0.20 [-0.39, -0.01]*** | **0.55 [0.22, 0.89]**** |
| Interval (3-4) × Envir cond (Sun) | **-0.24 [-0.43, -0.05]*** | **0.34 [0.01, 0.68]*** |

**Table S2.** Predicted values from the selected linear mixed models taking beetle species gain rate (see main text for definition) as the response, and the factors time interval (1-2, 2-3 and 3-4) and environmental conditions (sun-exposed or shaded) as predictors. There is a separate model for the following beetle functional groups: a) aspen specialists b) aspen generalists c) fungivores d) wood-feeders e) predators f) Saprophages/omnivores. Ninety-five % confidence intervals for the predictions are given in brackets.

| Predictor combination | a) Aspen specialists | b) Aspen generalists |
| --- | --- | --- |
| Interval 1-2 - Shade | 1.35 [1.00, 1.70] | 0.72 [0.64, 0.80] |
| Interval 2-3 - Shade | 1.31 [1.03, 1.60] | 0.42 [0.34, 0.50] |
| Interval 3-4 - Shade | 0.87 [0.63, 1.11] | 0.40 [0.32, 0.47] |
| Interval 1-2 - Sun | 1.24 [1.01, 1.46] | 0.66 [0.59, 0.74] |
| Interval 2-3 - Sun | 0.37 [0.15, 0.60] | 0.36 [0.29, 0.44] |
| Interval 3-4 - Sun | 0.42 [0.19, 0.64] | 0.34 [0.27, 0.42] |
|  | c) Fungivores | d) Wood-Feeders |
| Interval 1-2 - Shade | 0.72 [0.58, 0.85] | 0.40 [0.34, 0.47 |
| Interval 2-3 - Shade | 0.64 [0.50, 0.77] | 0.21 [0.15, 0.28] |
| Interval 3-4 - Shade | 0.64 [0.51, 0.78] | 0.22 [0.16, 0.28] |
| Interval 1-2 - Sun | 0.77 [0.64, 0.91] | 0.36 [0.30, 0.43] |
| Interval 2-3 - Sun | 0.39 [0.26, 0.52] | 0.17 [0.11, 0.23] |
| Interval 3-4 - Sun | 0.40 [0.27, 0.53] | 0.18 [0.12, 0.24] |
|  | e) Predators | f) Omnivores/saprophages |
| Interval 1-2 - Shade | 0.58 [0.48, 0.67] | 0.63 [0.46, 0.81] |
| Interval 2-3 - Shade | 0.49 [0.40, 0.59] | 0.07 [-0.10, 0.24] |
| Interval 3-4 - Shade | 0.42 [0.33, 0.52] | 0.38 [0.21, 0.55] |
| Interval 1-2 - Sun | 0.72 [0.63, 0.82] | 0.27 [0.11, 0.43] |
| Interval 2-3 - Sun | 0.44 [0.34, 0.53] | 0.26 [0.10, 0.42] |
| Interval 3-4 - Sun | 0.33 [0.24, 0.43] | 0.36 [0.20, 0.52] |

**Table S3.** Pairwise comparisons of species gain rates between exposed and shaded sites (exposed minus shaded) within each time interval. Comparisons are based on linear mixed models taking beetle species gain rate (see main text for definition) as the response, and the factors time interval (1-2, 2-3 and 3-4) and environmental conditions (sun-exposed or shaded) as predictors. There is a separate model for the following beetle functional groups: a) aspen specialists b) aspen generalists c) fungivores d) wood-feeders e) predators f) Saprophages/omnivores. Ninety-five % confidence intervals are given in brackets, and comparisons that are statistically significant on the 0.05 level are highlighted in bold. *** = P <= 0.001. ** = P <= 0.01. * = P <= 0.05. Note that the time × environment interaction was not significant for b) and d) so that the comparison for interval 1-2 is valid across all time intervals for these models.

| Time interval | a) Aspen specialists | b) Aspen generalists |
| --- | --- | --- |
| Interval 1-2 | -0.11 [-0.53, 0.31] | -0.05 [-0.13, 0.03] |
| Interval 2-3 | **-0.94 [-1.31, -0.58]***** | **-** |
| Interval 3-4 | **-0.45 [-0.78, -0.12]**** | **-** |
|  | c) Fungivores | d) Wood-Feeders |
| Interval 1-2 | 0.06 [-0.13, 0.24] | -0.04 [-0.10, 0.02] |
| Interval 2-3 | **-0.25 [-0.43, -0.06]*** | - |
| Interval 3-4 | **-0.25 [-0.43, -0.06]*** | - |
|  | e) Predators | f) Omnivores/saprophages |
| Interval 1-2 | **0.14 [0.01, 0.28]*** | **-0.36 [-0.60, -0.13]*** |
| Interval 2-3 | -0.06 [-0.19, 0.08] | 0.19 [-0.05, 0.43] |
| Interval 3-4 | -0.09 [-0.23, 0.04] | -0.02 [-0.25, 0.21] |

**Table S4.** Coefficients for the fixed effects in the selected linear mixed models taking beetle species loss rate (see main text for definition) as the response, and the factors time interval (1-2, 2-3 and 3-4) and environmental conditions (sun-exposed or shaded) as predictors. There is a separate model for the following beetle functional groups: a) aspen specialists b) aspen generalists c) fungivores d) wood-feeders e) predators f) Saprophages/omnivores. The intercept in all models represents shaded high stumps for the time interval 1-2. Ninety-five % confidence intervals are given in brackets, and terms that are statistically significant on the 0.05 level are highlighted in bold. *** = P <= 0.001. ** = P <= 0.01. * = P <= 0.05. Note that the time × environment interaction was not significant for d) and f) and was therefore dropped from these models.

| Parameter | a) Aspen specialists | b) Aspen generalists |
| --- | --- | --- |
| Intercept | **0.37 [0.08, 0.65]*** | **0.32 [0.25, 0.40]***** |
| Envir cond (Sun) | -0.23 [-0.57, 0.10] | -0.09 [-0.20, 0.01] |
| Interval (2-3) | -0.14 [-0.50, 0.23] | 0.06 [-0.05, 0.16] |
| Interval (3-4) | 0.16 [-0.19, 0.50] | -0.02 [-0.13, 0.08] |
| Interval (2-3) × Envir cond (Sun) | **0.63 [0.18, 1.07]**** | **0.15 [0.00, 0.29]*** |
| Interval (3-4) × Envir cond (Sun) | 0.18 [-0.25, 0.61] | **0.28 [0.14, 0.43]***** |
|  | c) Fungivores | d) Wood-Feeders |
| Intercept | **0.58 [0.47, 0.70]***** | **0.32 [0.24, 0.40]***** |
| Envir cond (Sun) | **-0.24 [-0.41, -0.08]**** | -0.04 [-0.12, 0.04] |
| Interval (2-3) | -0.03 [-0.19, 0.13] | 0.10 [0.00, 0.19] |
| Interval (3-4) | **-0.22 [-0.39, -0.06]**** | **0.13 [0.03, 0.23]*** |
| Interval (2-3) × Envir cond (Sun) | 0.22 [-0.01, 0.45] | **-** |
| Interval (3-4) × Envir cond (Sun) | **0.35 [0.12, 0.58]**** | - |
|  | e) Predators | f) Omnivores/saprophages |
| Intercept | **0.32 [0.22, 0.42]***** | **0.19 [0.05, 0.33]**** |
| Envir cond (Sun) | -0.05 [-0.19, 0.09] | 0.06 [-0.07, 0.19] |
| Interval (2-3) | 0.10 [-0.05, 0.24] | 0.15 [-0.01, 0.32] |
| Interval (3-4) | **0.16 [0.01, 0.30]*** | 0.05 [-0.11, 0.22] |
| Interval (2-3) × Envir cond (Sun) | **0.25 [0.04, 0.45]*** | **-** |
| Interval (3-4) × Envir cond (Sun) | 0.17 [-0.03, 0.37] | **-** |

**Table S5.** Predicted values from the selected linear mixed models taking beetle species loss rate (see main text for definition) as the response, and the factors time interval (1-2, 2-3 and 3-4) and environmental conditions (sun-exposed or shaded) as predictors. There is a separate model for the following beetle functional groups: a) aspen specialists b) aspen generalists c) fungivores d) wood-feeders e) predators f) Saprophages/omnivores. Ninety-five % confidence intervals for the predictions are given in brackets.

| Predictor combination | a) Aspen specialists | b) Aspen generalists |
| --- | --- | --- |
| Interval 1-2 - Shade | 0.37 [0.08, 0.65] | 0.32 [0.24, 0.41] |
| Interval 2-3 - Shade | 0.23 [0.00, 0.46] | 0.38 [0.29, 0.47] |
| Interval 3-4 - Shade | 0.52 [0.33, 0.72] | 0.36 [0.27, 0.45] |
| Interval 1-2 - Sun | 0.14 [-0.05, 0.32] | 0.23 [0.15, 0.32] |
| Interval 2-3 - Sun | 0.63 [0.44, 0.81] | 0.44 [0.35, 0.52] |
| Interval 3-4 - Sun | 0.47 [0.29, 0.65] | 0.49 [0.41, 0.58] |
|  | c) Fungivores | d) Wood-Feeders |
| Interval 1-2 - Shade | 0.58 [0.47, 0.70] | 0.32 [0.24, 0.40] |
| Interval 2-3 - Shade | 0.55 [0.43, 0.67] | 0.41 [0.33, 0.49] |
| Interval 3-4 - Shade | 0.36 [0.24, 0.47] | 0.45 [0.37, 0.53] |
| Interval 1-2 - Sun | 0.34 [0.22, 0.45] | 0.27 [0.20, 0.35] |
| Interval 2-3 - Sun | 0.53 [0.41, 0.64] | 0.37 [0.29, 0.45] |
| Interval 3-4 - Sun | 0.47 [0.35, 0.58] | 0.40 [0.32, 0.48] |
|  | e) Predators | f) Omnivores/saprophages |
| Interval 1-2 - Shade | 0.32 [0.22, 0.42] | 0.19 [0.05, 0.33] |
| Interval 2-3 - Shade | 0.42 [0.31, 0.52] | 0.34 [0.21, 0.48] |
| Interval 3-4 - Shade | 0.48 [0.38, 0.58] | 0.24 [0.10, 0.38] |
| Interval 1-2 - Sun | 0.27 [0.17, 0.37] | 0.25 [0.12, 0.38] |
| Interval 2-3 - Sun | 0.61 [0.51, 0.71] | 0.40 [0.27, 0.54] |
| Interval 3-4 - Sun | 0.60 [0.50, 0.70] | 0.30 [0.17, 0.43] |

**Table S6.** Pairwise comparisons between species loss rates at exposed and shaded sites (exposed minus shaded) within each time interval. Comparisons are based on linear mixed models taking beetle species loss rate (see main text for definition) as the response, and the factors time interval (1-2, 2-3 and 3-4) and environmental conditions (sun-exposed or shaded) as predictors. There is a separate model for the following beetle functional groups: a) aspen specialists b) aspen generalists c) fungivores d) wood-feeders e) predators f) Saprophages/omnivores. Ninety-five % confidence intervals are given in brackets, and comparisons that are statistically significant on the 0.05 level are highlighted in bold. *** = P <= 0.001. ** = P <= 0.01. * = P <= 0.05. Note that the time × environment interaction was not significant for d) and f) so that the comparison for interval 1-2 is valid across all time intervals for these models.

| Time interval | a) Aspen specialists | b) Aspen generalists |
| --- | --- | --- |
| Interval 1-2 | -0.23 [-0.57, 0.10] | -0.09 [-0.21, 0.03] |
| Interval 2-3 | **0.40 [0.10, 0.69]**** | 0.06 [-0.07, 0.18] |
| Interval 3-4 | -0.05 [-0.32, 0.21] | **0.13 [0.01, 0.26]*** |
|  | c) Fungivores | d) Wood-Feeders |
| Interval 1-2 | **-0.24 [-0.41, -0.08]**** | -0.04 [-0.12, 0.04] |
| Interval 2-3 | -0.02 [-0.19, 0.14] | - |
| Interval 3-4 | 0.11 [-0.05, 0.27] | - |
|  | e) Predators | f) Omnivores/saprophages |
| Interval 1-2 | -0.05 [-0.19, 0.09] | 0.06 [-0.07, 0.19] |
| Interval 2-3 | **0.20 [0.05, 0.34]*** | **-** |
| Interval 3-4 | 0.12 [-0.02, 0.26] | **-** |

**Table S7.** Coefficients for the fixed effects in the selected negative binomial mixed models taking beetle species richness as the response, and the factors year (1-4) and environmental conditions (sun-exposed or shaded) as predictors. There is a separate model for the following beetle functional groups: a) aspen specialists b) aspen generalists c) fungivores d) wood-feeders e) predators f) Saprophages/omnivores. The intercept in all models represents shaded high stumps in 2002 (i.e. year 1 of the succession). All estimates are on log scale. Ninety-five % confidence intervals are given in brackets, and terms that are statistically significant on the 0.05 level are highlighted in bold. *** = P <= 0.001. ** = P <= 0.01. * = P <= 0.05. Note that the time × environment interaction was not significant for d) and f) and was therefore dropped from these models.

| Parameter | a) Aspen specialists | b) Aspen generalists |
| --- | --- | --- |
| Intercept | **-1.24 [-1.87, -0.61]***** | **1.94 [1.76, 2.12]***** |
| Envir cond (Sun) | **1.43 [0.72, 2.13]***** | **0.43 [0.20, 0.67]***** |
| Year (2) | 0.51 [-0.27, 1.29] | **0.28 [0.07, 0.48]**** |
| Year (3) | **0.80 [0.05, 1.55]*** | **0.24 [0.03, 0.44]*** |
| Year (4) | **1.49 [0.81, 2.17]***** | **0.64 [0.44, 0.83]***** |
| Year (2) × Envir cond (Sun) | 0.69 [-0.17, 1.54] | 0.23 [-0.03, 0.49] |
| Year (3) × Envir cond (Sun) | -0.13 [-0.97, 0.71] | 0.14 [-0.13, 0.40] |
| Year (4) × Envir cond (Sun) | -0.61 [-1.38, 0.17] | -0.15 [-0.41, 0.11] |
|  | c) Fungivores | d) Wood-Feeders |
| Intercept | **1.03 [0.80, 1.27]***** | **-0.45 [-0.76, -0.13]**** |
| Envir cond (Sun) | **0.53 [0.22, 0.83]***** | **1.27 [0.95, 1.59]***** |
| Year (2) | 0.20 [-0.07, 0.47] | **0.48 [0.22, 0.74]***** |
| Year (3) | **0.30 [0.04, 0.57]*** | 0.14 [-0.14, 0.41] |
| Year (4) | **0.78 [0.54, 1.02]***** | **0.32 [0.05, 0.58]*** |
| Year (2) × Envir cond (Sun) | **0.46 [0.13, 0.79]**** | **-** |
| Year (3) × Envir cond (Sun) | 0.25 [-0.08, 0.57] | - |
| Year (4) × Envir cond (Sun) | -0.10 [-0.40, 0.21] | **-** |
|  | e) Predators | f) Omnivores/saprophages |
| Intercept | **1.01 [0.77, 1.25]***** | -0.08 [-0.35, 0.18] |
| Envir cond (Sun) | 0.25 [-0.07, 0.57] | **0.35 [0.12, 0.58]**** |
| Year (2) | **0.37 [0.09, 0.65]**** | 0.18 [-0.14, 0.50] |
| Year (3) | **0.41 [0.12, 0.69]**** | -0.03 [-0.36, 0.30] |
| Year (4) | **0.77 [0.51, 1.04]***** | 0.25 [-0.06, 0.57] |
| Year (2) × Envir cond (Sun) | 0.35 [-0.01, 0.72] | **-** |
| Year (3) × Envir cond (Sun) | 0.01 [-0.36, 0.38] | **-** |
| Year (4) × Envir cond (Sun) | -0.23 [-0.59, 0.12] | **-** |

**Table S8.** Predicted values from the selected negative binomial mixed models taking beetle species richness as the response, and the factors year (1-4) and environmental conditions (sun-exposed or shaded) as predictors. There is a separate model for the following beetle functional groups: a) aspen specialists b) aspen generalists c) fungivores d) wood-feeders e) predators f) Saprophages/omnivores. All predictions are back-transformed from log scale. Ninety-five % confidence intervals for the predictions are given in brackets.

| Predictor combination | a) Aspen specialists | b) Aspen generalists |
| --- | --- | --- |
| Year 1 - Shade | 0.29 [0.15, 0.54] | 6.96 [5.83, 8.32] |
| Year 2 - Shade | 0.48 [0.29, 0.81] | 9.20 [7.80, 10.85] |
| Year 3 - Shade | 0.64 [0.41, 1.02] | 8.81 [7.46, 10.41] |
| Year 4 - Shade | 1.29 [0.92, 1.81] | 13.18 [11.33, 15.34] |
| Year 1 - Sun | 1.21 [0.86, 1.70] | 10.74 [9.19, 12.54] |
| Year 2 - Sun | 4.01 [3.23, 4.98] | 17.84 [15.51, 20.52] |
| Year 3 - Sun | 2.36 [1.82, 3.06] | 15.58 [13.49, 17.98] |
| Year 4 - Sun | 2.93 [2.30, 3.72] | 17.47 [15.18, 20.11] |
|  | c) Fungivores | d) Wood-Feeders |
| Year 1 - Shade | 2.81 [2.22, 3.55] | 0.64 [0.47, 0.88] |
| Year 2 - Shade | 3.42 [2.73, 4.29] | 1.03 [0.77, 1.39] |
| Year 3 - Shade | 3.81 [3.06, 4.74] | 0.73 [0.54, 1.00] |
| Year 4 - Shade | 6.14 [5.08, 7.42] | 0.88 [0.65, 1.19] |
| Year 1 - Sun | 4.76 [3.90, 5.80] | 2.28 [1.75, 2.96] |
| Year 2 - Sun | 9.20 [7.77, 10.88] | 3.68 [2.92, 4.63] |
| Year 3 - Sun | 8.25 [6.94, 9.80] | 2.61 [2.03, 3.36] |
| Year 4 - Sun | 9.42 [7.97, 11.13] | 3.12 [2.46, 3.97] |
|  | e) Predators | f) Omnivores/saprophages |
| Year 1 - Shade | 2.73 [2.15, 3.47] | 0.92 [0.71, 1.20] |
| Year 2 - Shade | 3.96 [3.22, 4.88] | 1.10 [0.85, 1.43] |
| Year 3 - Shade | 4.10 [3.34, 5.03] | 0.89 [0.68, 1.18] |
| Year 4 - Shade | 5.93 [4.96, 7.10] | 1.19 [0.92, 1.52] |
| Year 1 - Sun | 3.52 [2.85, 4.36] | 1.31 [1.02, 1.68] |
| Year 2 - Sun | 7.27 [6.16, 8.58] | 1.57 [1.24, 1.99] |
| Year 3 - Sun | 5.32 [4.43, 6.39] | 1.27 [0.98, 1.65] |
| Year 4 - Sun | 6.05 [5.08, 7.21] | 1.69 [1.34, 2.12] |

**Table S9.** Pairwise comparisons between species richness at exposed and shaded sites (exposed minus shaded) within each year. Comparisons are based on negative binomial mixed models taking beetle species richness as the response, and the factors year (1-4) and environmental conditions (sun-exposed or shaded) as predictors. There is a separate model for the following beetle functional groups: a) aspen specialists b) aspen generalists c) fungivores d) wood-feeders e) predators f) Saprophages/omnivores. Ninety-five % confidence intervals are given in brackets, and comparisons that are statistically significant on the 0.05 level are highlighted in bold. *** = P <= 0.001. ** = P <= 0.01. * = P <= 0.05. All comparisons are on the log scale. Note that the time × environment interaction was not significant for d) and f) so that the comparison for year 1 is valid across all years for these models.

| Year | a) Aspen specialists | b) Aspen generalists |
| --- | --- | --- |
| Year 1 | **1.43 [0.72, 2.13]***** | **0.43 [0.20, 0.67]***** |
| Year 2 | **2.12 [1.56, 2.68]***** | **0.66 [0.45, 0.88]***** |
| Year 3 | **1.30 [0.77, 1.82]***** | **0.57 [0.35, 0.79]***** |
| Year 4 | **0.82 [0.41, 1.23]***** | **0.28 [0.08, 0.49]**** |
|  | c) Fungivores | d) Wood-Feeders |
| Year 1 | **0.53 [0.22, 0.83]***** | **1.27 [0.95, 1.59] ***** |
| Year 2 | **0.99 [0.71, 1.27] ***** | - |
| Year 3 | **0.77 [0.49, 1.05] ***** | - |
| Year 4 | **0.43 [0.18, 0.68] ***** | - |
|  | e) Predators | f) Omnivores/saprophages |
| Year 1 | 0.25 [-0.07, 0.57] | **0.35 [0.12, 0.58] **** |
| Year 2 | **0.61 [0.34, 0.87] ***** | **-** |
| Year 3 | 0.26 [-0.01, 0.53] | **-** |
| Year 4 | 0.02 [-0.23, 0.27] | **-** |

**Table S10.** Coefficients for the fixed effects in the selected negative binomial mixed models taking beetle abundance as the response, and the factors year (1-4) and environmental conditions (sun-exposed or shaded) as predictors. There is a separate model for the following beetle functional groups: a) aspen specialists b) aspen generalists c) fungivores d) wood-feeders e) predators f) Saprophages/omnivores. The intercept in all models represents shaded high stumps in 2002 (i.e. year 1 of the succession). All estimates are on log scale. Ninety-five % confidence intervals are given in brackets, and terms that are statistically significant on the 0.05 level are highlighted in bold. *** = P <= 0.001. ** = P <= 0.01. * = P <= 0.05. Note that the time × environment interaction was not significant for d) and f) and was therefore dropped from these models.

| Parameter | a) Aspen specialists | b) Aspen generalists |
| --- | --- | --- |
| Intercept | **-0.99 [-1.68, -0.31]**** | **2.48 [2.23, 2.72]***** |
| Envir cond (Sun) | **1.34 [0.51, 2.17]**** | **0.85 [0.52, 1.18]***** |
| Year (2) | 0.23 [-0.57, 1.03] | **0.33 [0.06, 0.60]*** |
| Year (3) | **1.23 [0.48, 1.98]**** | **0.50 [0.23, 0.77]***** |
| Year (4) | **2.24 [1.53, 2.95]***** | **1.32 [1.06, 1.58]***** |
| Year (2) × Envir cond (Sun) | **1.78 [0.83, 2.73]***** | **0.40 [0.04, 0.76]*** |
| Year (3) × Envir cond (Sun) | 0.61 [-0.30, 1.53] | -0.06 [-0.42, 0.30] |
| Year (4) × Envir cond (Sun) | -0.68 [-1.55, 0.20] | **-0.67 [-1.03, -0.32]***** |
|  | c) Fungivores | d) Wood-Feeders |
| Intercept | **1.40 [1.07, 1.72]***** | -0.27 [-0.68, 0.13] |
| Envir cond (Sun) | **0.58 [0.14, 1.02]**** | **2.25 [1.78, 2.72]***** |
| Year (2) | 0.24 [-0.15, 0.63] | **0.65 [0.39, 0.91]***** |
| Year (3) | **0.97 [0.59, 1.34] ***** | 0.14 [-0.13, 0.41] |
| Year (4) | **1.64 [1.27, 2.01] ***** | 0.25 [-0.02, 0.52] |
| Year (2) × Envir cond (Sun) | **0.89 [0.38, 1.40] ***** | **-** |
| Year (3) × Envir cond (Sun) | 0.19 [-0.31, 0.70] | **-** |
| Year (4) × Envir cond (Sun) | -0.39 [-0.89, 0.10] | **-** |
|  | e) Predators | f) Omnivores/saprophages |
| Intercept | **1.65 [1.34, 1.96]***** | **0.57 [0.27, 0.87]***** |
| Envir cond (Sun) | 0.28 [-0.15, 0.72] | **1.09 [0.74, 1.43]***** |
| Year (2) | 0.31 [-0.03, 0.66] | **0.45 [0.22, 0.69]***** |
| Year (3) | 0.34 [-0.01, 0.68] | -0.04 [-0.29, 0.20] |
| Year (4) | **1.40 [1.07, 1.73]***** | **0.40 [0.16, 0.63]***** |
| Year (2) × Envir cond (Sun) | **0.70 [0.23, 1.17]**** | **-** |
| Year (3) × Envir cond (Sun) | 0.22 [-0.25, 0.69] | **-** |
| Year (4) × Envir cond (Sun) | **-0.58 [-1.04, -0.12]*** | **-** |

**Table S11.** Predicted values from the selected negative binomial mixed models taking beetle abundance as the response, and the factors year (1-4) and environmental conditions (sun-exposed or shaded) as predictors. There is a separate model for the following beetle functional groups: a) aspen specialists b) aspen generalists c) fungivores d) wood-feeders e) predators f) Saprophages/omnivores. All predictions are back-transformed from log scale. Ninety-five % confidence intervals for the predictions are given in brackets.

| Predictor combination | a) Aspen specialists | b) Aspen generalists |
| --- | --- | --- |
| Year 1 - Shade | 0.37 [0.19, 0.73] | 11.91 [9.34, 15.19] |
| Year 2 - Shade | 0.47 [0.25, 0.88] | 16.56 [13.08, 20.97] |
| Year 3 - Shade | 1.27 [0.76, 2.13] | 19.66 [15.57, 24.83] |
| Year 4 - Shade | 3.47 [2.21, 5.47] | 44.55 [35.54, 55.86] |
| Year 1 - Sun | 1.41 [0.86, 2.30] | 27.81 [22.20, 34.82] |
| Year 2 - Sun | 10.49 [6.99, 15.75] | 57.71 [46.35, 71.84] |
| Year 3 - Sun | 8.94 [5.90, 13.54] | 43.19 [34.62, 53.88] |
| Year 4 - Sun | 6.74 [4.45, 10.20] | 53.11 [42.64, 66.14] |
|  | c) Fungivores | d) Wood-Feeders |
| Year 1 - Shade | 4.05 [2.92, 5.61] | 0.76 [0.51, 1.14] |
| Year 2 - Shade | 5.15 [3.76, 7.06] | 1.46 [0.98, 2.15] |
| Year 3 - Shade | 10.64 [7.93, 14.29] | 0.87 [0.58, 1.30] |
| Year 4 - Shade | 20.84 [15.69, 27.69] | 0.97 [0.65, 1.45] |
| Year 1 - Sun | 7.26 [5.40, 9.78] | 7.19 [5.08, 10.19] |
| Year 2 - Sun | 22.52 [17.08, 29.69] | 13.78 [9.86, 19.26] |
| Year 3 - Sun | 23.13 [17.52, 30.55] | 8.25 [5.85, 11.63] |
| Year 4 - Sun | 25.22 [19.17, 33.18] | 9.20 [6.52, 12.99] |
|  | e) Predators | f) Omnivores/saprophages |
| Year 1 - Shade | 5.21 [3.80, 7.12] | 1.76 [1.30, 2.39] |
| Year 2 - Shade | 7.11 [5.25, 9.62] | 2.78 [2.08, 3.71] |
| Year 3 - Shade | 7.29 [5.40, 9.86] | 1.69 [1.25, 2.29] |
| Year 4 - Shade | 21.17 [15.95, 28.11] | 2.62 [1.96, 3.50] |
| Year 1 - Sun | 6.92 [5.13, 9.34] | 5.24 [3.97, 6.91] |
| Year 2 - Sun | 19.03 [14.40, 25.14] | 8.25 [6.32, 10.76] |
| Year 3 - Sun | 12.14 [9.13, 16.13] | 5.03 [3.81, 6.64] |
| Year 4 - Sun | 15.82 [11.95, 20.94] | 7.79 [5.95, 10.20] |

**Table S12.** Pairwise comparisons between abundance at exposed and shaded sites (exposed minus shaded) within each year. Comparisons are based on negative binomial mixed models taking beetle abundance as the response, and the factors year (1-4) and environmental conditions (sun-exposed or shaded) as predictors. There is a separate model for the following beetle functional groups: a) aspen specialists b) aspen generalists c) fungivores d) wood-feeders e) predators f) Saprophages/omnivores. Ninety-five % confidence intervals are given in brackets, and comparisons that are statistically significant on the 0.05 level are highlighted in bold. *** = P <= 0.001. ** = P <= 0.01. * = P <= 0.05. All comparisons are on the log scale. Note that the time × environment interaction was not significant for d) and f) so that the comparison for year 1 is valid across all years for these models.

| Year | a) Aspen specialists | b) Aspen generalists |
| --- | --- | --- |
| Year 1 | **1.34 [0.51, 2.17]**** | **0.85 [0.52, 1.18]***** |
| Year 2 | **3.11 [2.37, 3.86]***** | **1.25 [0.93, 1.57]***** |
| Year 3 | **1.95 [1.30, 2.61]***** | **0.79 [0.47, 1.11]***** |
| Year 4 | **0.66 [0.05, 1.28]*** | 0.18 [-0.14, 0.49] |
|  | c) Fungivores | d) Wood-Feeders |
| Year 1 | **0.58 [0.14, 1.02]**** | **2.25 [1.78, 2.72]***** |
| Year 2 | **1.48 [1.06, 1.89]***** | **-** |
| Year 3 | **0.78 [0.37, 1.18]***** | **-** |
| Year 4 | 0.19 [-0.20, 0.58] | **-** |
|  | e) Predators | f) Omnivores/saprophages |
| Year 1 | 0.28 [-0.15, 0.72] | **1.09 [0.74, 1.43]***** |
| Year 2 | **0.98 [0.57, 1.39]***** | **-** |
| Year 3 | **0.51 [0.09, 0.92]*** | **-** |
| Year 4 | -0.29 [-0.69, 0.11] | **-** |

**Table S13.** Coefficients for the fixed effects in the negative binomial mixed models taking beetle species richness (a) and abundance (b) as the response, and the factors year (1-2), environmental conditions (sun-exposed or shaded) and functional group (specialist or generalist) as predictors. The intercept in the models represent shaded high stumps in 2002 (i.e. year 1 of the succession) for generalists. All estimates are on log scale. Ninety-five % confidence intervals are given in brackets, and terms that are statistically significant on the 0.05 level are highlighted in bold. *** = P <= 0.001. ** = P <= 0.01. * = P <= 0.05.

| Parameter | a) Species richness | b) Abundance |
| --- | --- | --- |
| Intercept | **1.89 [1.70, 2.09]***** | **2.40 [2.11, 2.69]***** |
| Envir cond (Sun) | **0.46 [0.20, 0.72]***** | **0.92 [0.53, 1.32]***** |
| Year (2) | **0.28 [0.10, 0.47]**** | **0.36 [0.07, 0.66]*** |
| Group (Specialist) | **-3.15 [-3.82, -2.48]***** | **-3.22 [-3.79, -2.65]***** |
| Year (2) × Envir cond (Sun) | 0.23 [-0.01, 0.46] | 0.38 [-0.02, 0.78] |
| Group (Specialist) × Envir cond (Sun) | **0.98 [0.23, 1.73]*** | 0.32 [-0.37, 1.00] |
| Year (2) × Group (Specialist) | 0.23 [-0.62, 1.07] | -0.13 [-0.90, 0.63] |
| Year (2) × Group (Specialist) × Envir cond (Sun) | 0.46 [-0.47, 1.40] | **1.41 [0.51, 2.31]**** |

**Table S14.** Predicted values from the negative binomial mixed models taking beetle species richness (a) and abundance (b) as the response, and the factors year (1-2), environmental conditions (sun-exposed or shaded) and functional group (specialist or generalist) as predictors. All predictions are back-transformed from log scale. Ninety-five % confidence intervals for the predictions are given in brackets.

| Predictor combination | a) Species richness | b) Abundance |
| --- | --- | --- |
| Specialist - Year 1 - Shade | 0.28 [0.23, 0.35] | 0.44 [0.33, 0.59] |
| Specialist - Year 2 - Shade | 0.47 [0.40, 0.57] | 0.55 [0.42, 0.73] |
| Specialist - Year 1 - Sun | 1.20 [1.01, 1.43] | 1.52 [1.17, 1.99] |
| Specialist - Year 2 - Sun | 3.99 [3.40, 4.68] | 11.52 [8.86, 14.97] |
| Generalist - Year 1 - Shade | 6.63 [3.40, 12.93] | 11.02 [6.25, 19.45] |
| Generalist - Year 2 - Shade | 8.81 [5.21, 14.89] | 15.82 [9.37, 26.72] |
| Generalist - Year 1 - Sun | 10.55 [7.47, 14.90] | 27.78 [18.99, 40.64] |
| Generalist - Year 2 - Sun | 17.55 [14.07, 21.89] | 58.37 [44.11, 77.24] |


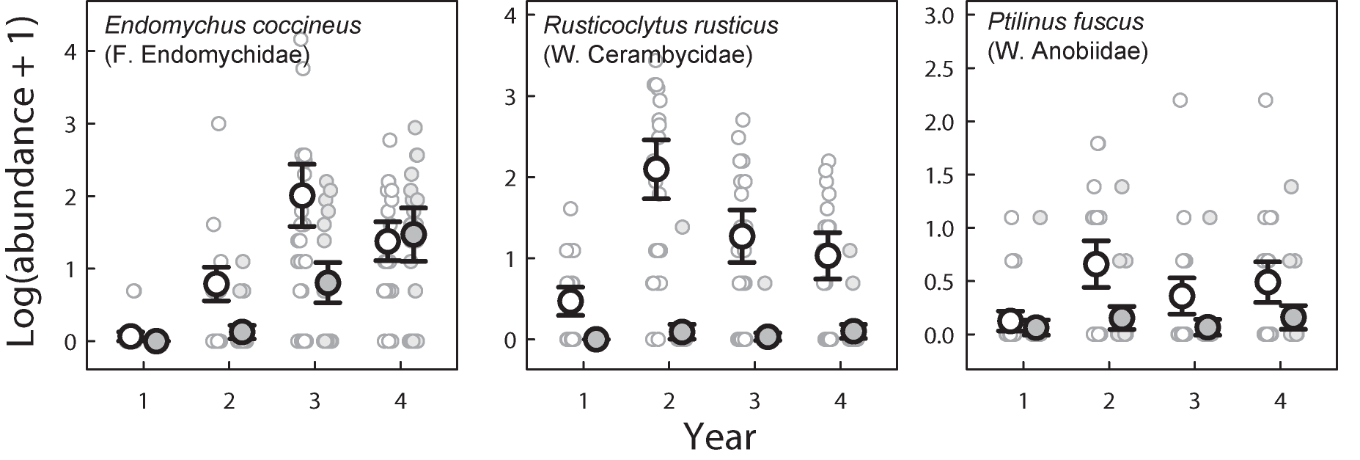


**Figure S1**. Variation in the log transformed abundances of common aspen specialist beetle species across the study years in sun-exposed (white symbols) and shaded (grey symbols) sites. Large symbols: mean abundances for each combination of year and environmental conditions. Error bars represent 95 % confidence intervals. Small symbols: original datapoints, i.e. the observed counts for individuals traps. The species that are shown collectively constitute at least 75 % of the total individual counts for their respective trophic guilds. The family and trophic guild of each species are given in parenthesis. F = Fungivore. W = Wood-feeding. Note that the range of the Y axis varies between panels.


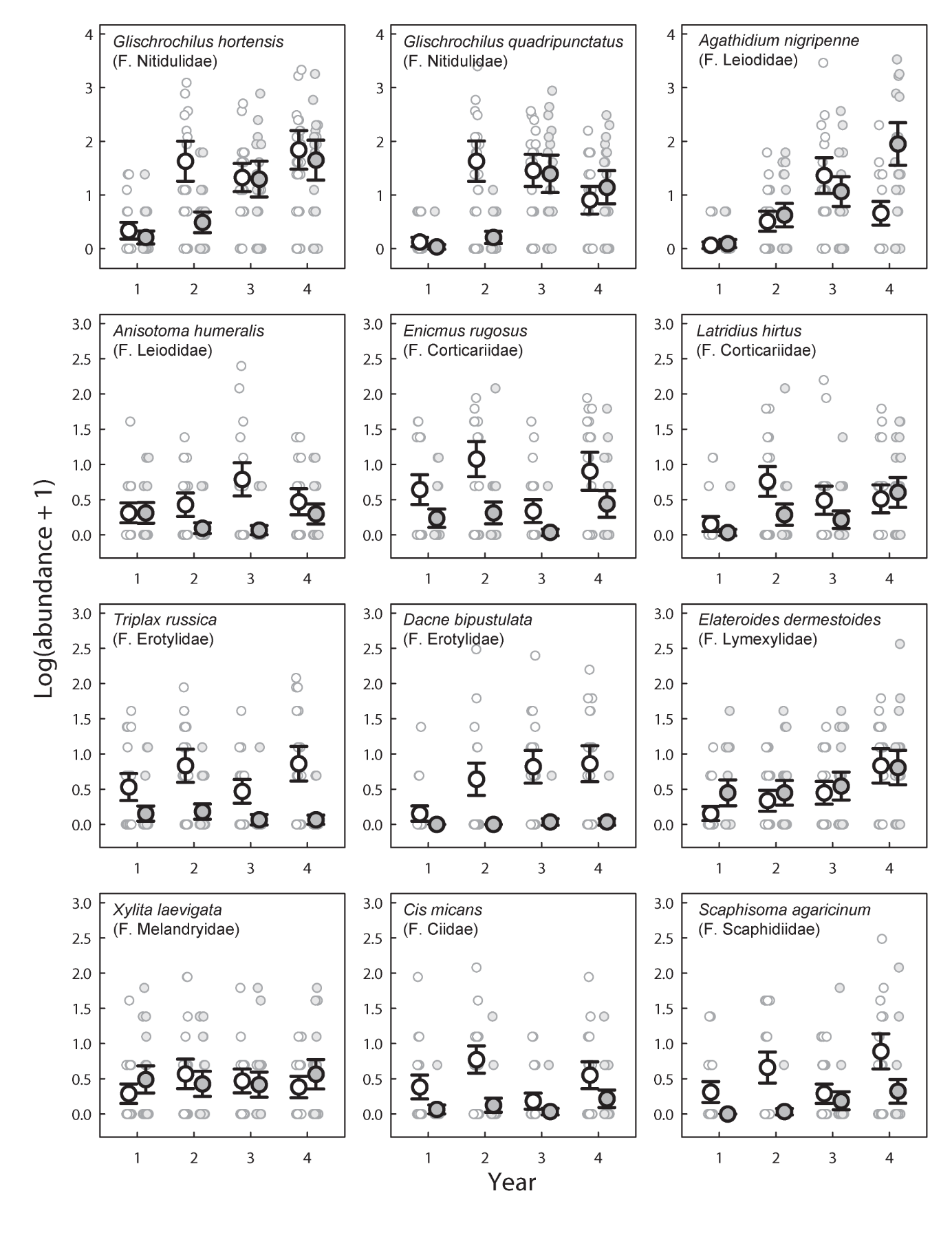


**Figure S2** (Part 1. Caption on next page).


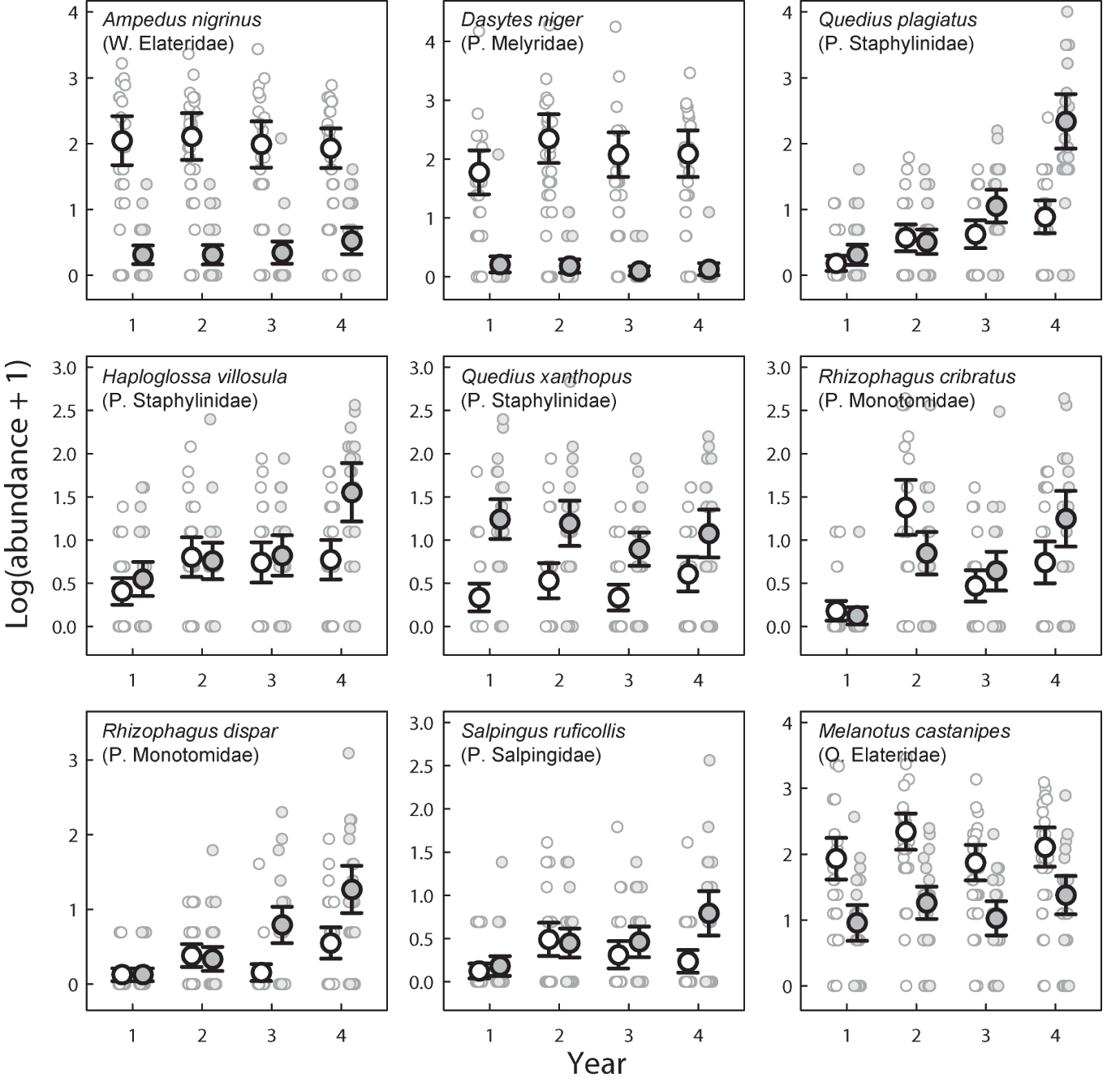


**Figure S2** (Part 2). Variation in the log transformed abundances of common aspen generalist beetle species across the study years in sun-exposed (white symbols) and shaded (grey symbols) sites. Large symbols: mean abundances for each combination of year and environmental conditions. Error bars represent 95 % confidence intervals. The species that are shown collectively constitute at least 75 % of the total individual counts for their respective trophic guilds. Small symbols: original datapoints, i.e. the observed counts for individuals traps. The family and trophic guild of each species are given in parenthesis. F = Fungivore. W = Wood-feeding. P = predator. O = Other. Note that the range of the Y axis varies between panels.
